# Medicaid Expansion and Buprenorphine Dispensing in Early vs Recent Expansion States

**DOI:** 10.1001/jamanetworkopen.2025.59803

**Published:** 2026-02-18

**Authors:** Nicole Siegal, Sumedha Gupta, Jennifer Miles, Hillary Samples, Kosali Simon, Matthew C. Aalsma, Stephen Crystal

**Affiliations:** 1 O’Neill School of Public and Environmental Affairs, Indiana University, Bloomington; 2 Department of Economics, Indiana University Indianapolis, Indianapolis; 3 Center for Health Services Research, Rutgers Institute for Health, Health Care Policy and Aging Research, Rutgers University, New Brunswick, New Jersey; 4 Department of Family Medicine and Community Health, Rutgers Robert Wood Johnson Medical School, New Brunswick, New Jersey; 5 Center for Pharmacoepidemiology and Treatment Science, Rutgers Institute for Health, Health Care Policy and Aging Research, Rutgers University, New Brunswick, New Jersey; 6 Rutgers School of Public Health, Piscataway, New Jersey; 7 National Bureau of Economic Research, Cambridge, Massachusetts; 8 Children’s Health Services Research Division, Department of Pediatrics, Indiana University School of Medicine, Indianapolis; 9 School of Social Work, Rutgers University, New Brunswick, New Jersey

## Abstract

**Importance:**

Patient Protection and Affordable Care Act (ACA) Medicaid expansion has been linked to improved uptake of medications for opioid use disorder (MOUD) access for Medicaid enrollees, but evidence is limited on the associations of post-2018 expansions with population-level treatment. Moreover, prior studies have not implemented modern staggered-adoption estimators or examined outcomes by payer type, risking biased and incomplete inference.

**Objectives:**

To evaluate the association of Medicaid expansion with buprenorphine treatment rates (overall and by payer) across all expansion states using updated data and methods and to test whether post-2018 expansions (post–X-waiver and telehealth era) differed from earlier ones.

**Design, Setting, and Participants:**

Cross-sectional study of buprenorphine dispensing in participants aged 18 years or older from 2013 to 2024 using the IQVIA Longitudinal Prescription Database containing over 90% of US retail pharmacy claims across 51 US jurisdictions (50 states plus Washington, DC). Cross-sectional difference-in-differences analysis leveraging staggered adoption of changes in monthly buprenorphine MOUD dispensing rates associated with Medicaid expansion.

**Exposure:**

Medicaid expansion under the ACA, implemented state-by-state, covering 41 expansion states (plus DC) and 10 nonexpansion states, measured by state and month.

**Main Outcomes and Measures:**

The primary outcome was the state-month rate of unique individuals receiving 1 or more buprenorphine prescriptions for opioid use disorder per 100 000 residents (all-payer). Secondary outcomes were payer-specific rates—Medicaid, commercial, Medicare, and self-pay—each per 100 000 persons in the corresponding insured population.

**Results:**

As the first year of any expansion is 2014, 2013 served as the baseline year. In 2013, there were 659 046 unique patients, 58.2% were male, and the mean (SD) age was 36.6 (0.02) years. For 149 648 295 dispensations for 4 596 264 unique patients across 41 expansion states (including DC) and 10 nonexpansion states from 2013 to 2024, Medicaid expansion increased total buprenorphine treatment rates only in states expanding in 2019 or later (21.1% relative increase; average treatment effect on the treated, 28.67 per 100 000; 95% CI, 8.20-49.15 per 100 000). Gains were most pronounced in high-need recent expanders, such as Maine (57.22 per 100 000; 95% CI, 44.20-70.24 per 100 000), Virginia (44.46 per 100 000; 95% CI, 31.44-57.49 per 100 000), and Oklahoma (22.98 per 100 000; 95% CI, 12.84-33.12 per 100 000).

**Conclusions and Relevance:**

In this cross-sectional study using contemporary data and methods, post-2018 Medicaid expansions were associated with substantial increases in buprenorphine treatment, particularly in high-need states operating under newer prescribing regulations. These findings underscore Medicaid expansion’s role in improving opioid use disorder treatment access and can inform state and federal efforts to expand evidence-based care.

## Key Points

**Question:**

How did population-level (all-payer) buprenorphine dispensing for opioid use disorder change following Medicaid expansion, and did post-2018 expansions differ?

**Findings:**

In this cross-sectional study of 149 648 295 dispensations for 4 596 264 unique patients, difference-in-differences analysis (2013-2024) found that Medicaid expansion was associated with a 21.1% relative increase in all-payer buprenorphine dispensing in recent (2019-2023) but not earlier (2014-2016) state expanders.

**Meaning:**

These findings suggest that amid policy and practice changes, Medicaid expansions were associated with improved opioid use disorder treatment access, and despite financial pressures on the sustainability of these expansions from reconciliation legislation, Medicaid access remains central to national opioid overdose epidemic response.

## Teaser

This cross-sectional study evaluates the association of Medicaid expansion with buprenorphine treatment rates and tests whether post-2018 expansions differed from earlier ones.

## Introduction

The US remains in a severe opioid overdose crisis, with over 100 000 drug overdose deaths annually in recent years; approximately two-thirds involved opioids.^1^ While provisional 2024 data indicate declines from pandemic-era peaks, more than 80 000 overdose deaths still occurred.^2^ Expanding access to effective treatment for opioid use disorder (OUD) remains a public health priority. Medicaid finances over half of medications for OUD (MOUD) nationally.^3^ Yet, many states have not adopted the Patient Protection and Affordable Care Act’s (ACA) Medicaid expansion, leaving substantial numbers of low-income adults without treatment coverage.^4^

Among FDA-approved MOUD, buprenorphine is especially important given its proven efficacy and availability in general medical settings.^5^ However, access remains limited and uneven, with substantial geographic and socioeconomic disparities.^6^ Medicaid expansion may reduce financial barriers to treatment access, but it remains unclear whether observed increases reflect true gains in treatment or shifts from other sources of payment (crowd-out from private or self-pay). Because individuals with opioid use disorder are disproportionately single adults eligible for expansion, 61% in 1 study,^7^ understanding expansion’s impact on treatment access is essential.

Despite substantial prior research, important gaps remain. Most studies analyzed data only through 2018, omitting recent Medicaid expansions and changes in the treatment landscape. For example, Golan et al^6^ reported reduced income-related disparities in buprenorphine treatment after expansion in urban counties, but their analysis ended in 2018, did not distinguish payers, and did not apply newer causal inference methods. Similarly, Olfson et al^8^ and Knudsen et al^9^ found postexpansion increases in Medicaid-paid buprenorphine prescriptions using traditional difference-in-differences (DiD) designs that may be biased under staggered policy adoption. Because prior work largely examined early adopting states, the effects of more recent expansions implemented in different policy and clinical contexts remain uncertain (see eAppendix 1 in Supplement 1 for a detailed review).

In this study, we leverage a national all-payer prescription claims database (2013-2024) to examine the association between Medicaid expansion and buprenorphine treatment rates. We applied modern DiD methods that account for staggered adoption timing and cohort-specific effects.^10^ We addressed 3 questions: (1) how overall (all-payer) buprenorphine treatment rates changed after expansion; (2) whether outcomes differed between early (2014-2016) and more recent (2019-2023) expansions; and (3) how outcomes varied by payer (Medicaid, commercial, Medicare, or self-pay).

## Methods

This retrospective, cross-sectional study used deidentified secondary data and was deemed exempt from informed consent by the Indiana University institutional review board. The study followed the Strengthening the Reporting of Observational Studies in Epidemiology (STROBE) reporting guideline for cross-sectional studies.^11^

### Data Source and Sample

We used the IQVIA Longitudinal Prescription Database (LRx), a national all-payer pharmacy claims dataset covering approximately 92% of prescriptions dispensed at US retail, mail-order, and long-term care pharmacies. LRx includes deidentified patient and prescriber identifiers, prescription details, and payer type (Medicaid, Medicare, commercial, or cash or self-pay). We extracted all buprenorphine dispensations for OUD from 2013 to 2024 and aggregated them to the state-month level.

Medicaid expansion status and timing were obtained from the Kaiser Family Foundation (KFF). The sample included 41 expansion states (including DC) and 10 nonexpansion states. Early adopters expanded in 2014 to 2016 and recent adopters in 2019 to 2023; 25 states expanded on January 1, 2014, 12 more by 2016, and 9 more between 2019 and 2023 (eTable 1 in Supplement 1). Preperiod opioid overdose mortality was measured using 2012 National Center for Health Statistics National Vital Statistics System data, aggregated to the state-year level using *International Statistical Classification of Diseases and Related Health Problems, Tenth Revision* codes T40.0 to T40.6.

### Outcome Measures

The primary outcome was the monthly all-payer rate of dispensed buprenorphine prescriptions, defined as the unique individuals per state-month filling 1 or more buprenorphine prescription per 100 000 state residents using US Census state population estimates. This measure reflects treatment initiation and continuation rather than prescribing decisions. Secondary outcomes were payer-specific rates, calculated analogously for Medicaid, commercial insurance, Medicare, or cash or self-pay (a proxy for uninsured). Payer-specific population denominators were obtained from KFF State Health Facts (2008-2023), based on aggregated American Community Survey data.^12^ Standardization per 100 000 population enabled comparisons across states and over time.

### Study Design

We evaluated changes in buprenorphine prescription-dispensation rates associated with Medicaid expansion using a DiD framework. To account for staggered adoption, we applied the Callaway and Sant’Anna estimator, which estimates group- and time-specific effects and avoids biases in conventional 2-way fixed-effects models.^10^ Postexpansion outcomes in adopting states were compared with counterfactual trends from never-expanded states, rather than assuming a single homogeneous effect across all states and time periods. Models included state fixed effects (to adjust for time-invariant state differences) and time fixed effects (to adjust for national shocks and trends). Analyses were stratified by adoption timing (early [2014-2016] vs recent [2019-2023]) and by baseline opioid overdose mortality rates (above vs below the national median). Full description of the model is in eAppendix 2 in Supplement 1.

### Statistical Analysis

For each expansion cohort, we estimated the average treatment effect on the treated (ATT), defined as the change in buprenorphine treatment rates attributable to Medicaid expansion relative to the counterfactual of no expansion. ATT estimates are reported as absolute changes per 100 000 population and as percentage changes from the pre-expansion baseline mean. We evaluated the parallel trends assumption using event-study analyses, estimating cohort-specific changes before and after expansion and examining pre-expansion coefficients to assess baseline differences (reported in eAppendix 2 in Supplement 1).

Robustness checks included (1) using not-yet treated states as controls until their expansion date; (2) alternative staggered DiD specifications; (3) leave-one-out analysis of control states; and (4) falsification tests among control states. Statistical significance was defined as a 2-sided *P* < .05, with state-clustered SEs. Analyses were conducted using Stata version 18 (StataCorp) and R version 4.5 (R Foundation for Statistical Computing).

## Results

### Description of Sample

Our sample included 149 648 295 prescription dispensations for 4 596 264 unique patients nationwide from 2013 to 2024. As the first year of any expansion is 2014, 2013 served as the baseline year. In 2013, there were 659 046 unique patients, 41.8% were female, the mean (SD) age was 36.6 (0.02) years, 9.6% were younger than 25 years, and 76.2% were aged 25 to 49 years.

### Overall Associations of Medicaid Expansion With Buprenorphine Treatment

**Figure 1.**
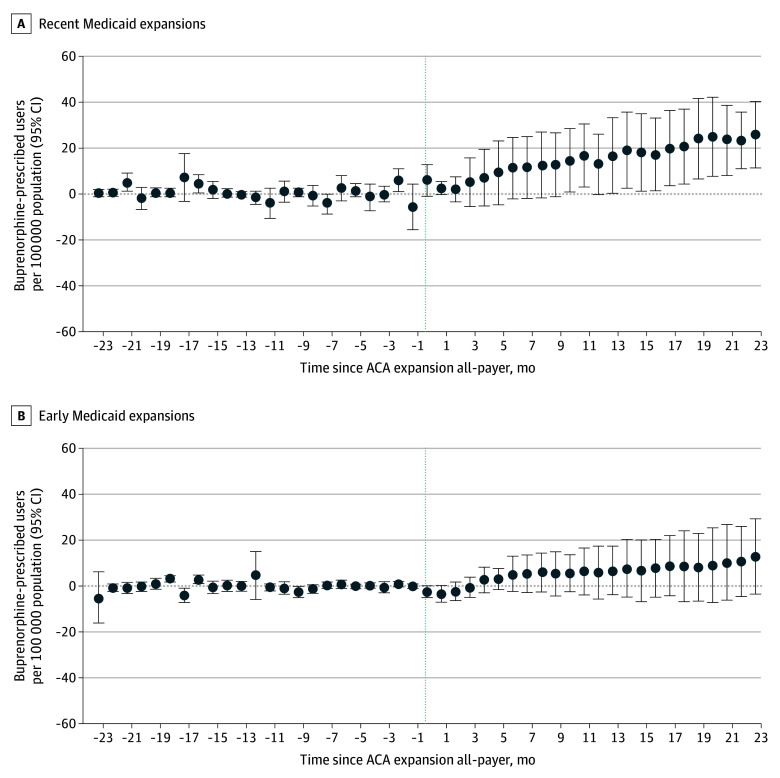
Difference-in-Differences Plot of Medicaid Expansions and Buprenorphine Patients—All-Payer A staggered adoption difference-in-differences event study plot, covering 24 months before and 24 months after the start of each Medicaid expansion, was used to evaluate the rate of buprenorphine prescribed users per 100 000 of population in each state-month cell. Never-treated states were used as the control group. Data are from IQVIA longitudinal prescription database for 2013 to 2024, aggregating prescriptions for buprenorphine by state and month. Data for expansion dates from the Kaiser Family Foundation and state populations from US Census Bureau. The treatment group analysis is limited in Figure 1A to early adopters (expanded in 2014-2016) and Figure 1B to recent adopters (2019-2023; see eTable 1 in the Supplement). ACA indicates Patient Protection and Affordable Care Act.

Medicaid expansion was associated with a significant increase in buprenorphine treatment rates among states that expanded in 2019 or later, whereas outcomes in earlier-adopting states were smaller and not statistically significant. As shown in Figure 1, recent-expansion states experienced a postexpansion increase of 28.67 (95% CI, 8.20-49.15) per 100 000 population (a 21.1% increase over baseline) compared with nonexpansion states. In contrast, states that expanded in 2014 to 2016 had an estimated increase of 15.25 (95% CI, –3.88 to 34.37) per 100 000 (13.4% above baseline), which was not statistically distinguishable from 0. Event-study analyses confirmed parallel pre-expansion trends between expansion and comparison states (Figure 1 and eFigure 2 in Supplement 1), supporting the interpretation of our results. Results were also similar when stratified by baseline opioid overdose mortality, with no differential outcomes observed between states with high vs low pre-expansion death rates (eFigure 3 in Supplement 1). Baseline buprenorphine treatment levels were higher in early expansion states than in recent-expansion states (141.8 vs 97.2 per 100 000 residents), providing context for the smaller relative increases observed among early adopters.

In contrast, a conventional 2-way fixed-effects DiD model that ignored staggered adoption detected no significant overall association (eFigure 2 in Supplement 1), underscoring the importance of modern estimators. Results were robust to using not-yet treated states as the control group (eFigure1 in Supplement 1), exclusion of individual control states (eFigure 8 in Supplement 1), and falsification tests among control states showed no spurious effects (eFigure 9 in Supplement 1).

### Payer-Specific Outcomes

Increases in buprenorphine treatment following Medicaid expansion were concentrated among Medicaid beneficiaries. Early expansion states experienced an increase of 44.19 Medicaid buprenorphine patients per 100 000 enrollees (37.5% increase from baseline of 117.88 per 100 000), whereas recent-expansion states saw a much larger increase of 206.4 per 100 000 (90.7% increase from baseline of 227.49 per 100 000) (eFigure 4 in Supplement 1). These Medicaid-specific gains were significant, although only recent expansions were associated with a significant all-payer increase.

By contrast, recent-expansion states showed modest declines in commercially insured buprenorphine patients (9.6% decline from baseline) (eFigure 5 in Supplement 1), suggesting some substitution from private to Medicaid coverage. For recent-expanders, no significant changes were observed in self-pay or Medicare-financed treatment (eFigures 6-7 in Supplement 1). Taken together, these results suggest that Medicaid expansion primarily increased treatment by expanding Medicaid coverage, with limited payer substitution and a net positive association with overall treatment, especially in recently adopting states.

### State-Specific Variation

**Figure 2.**
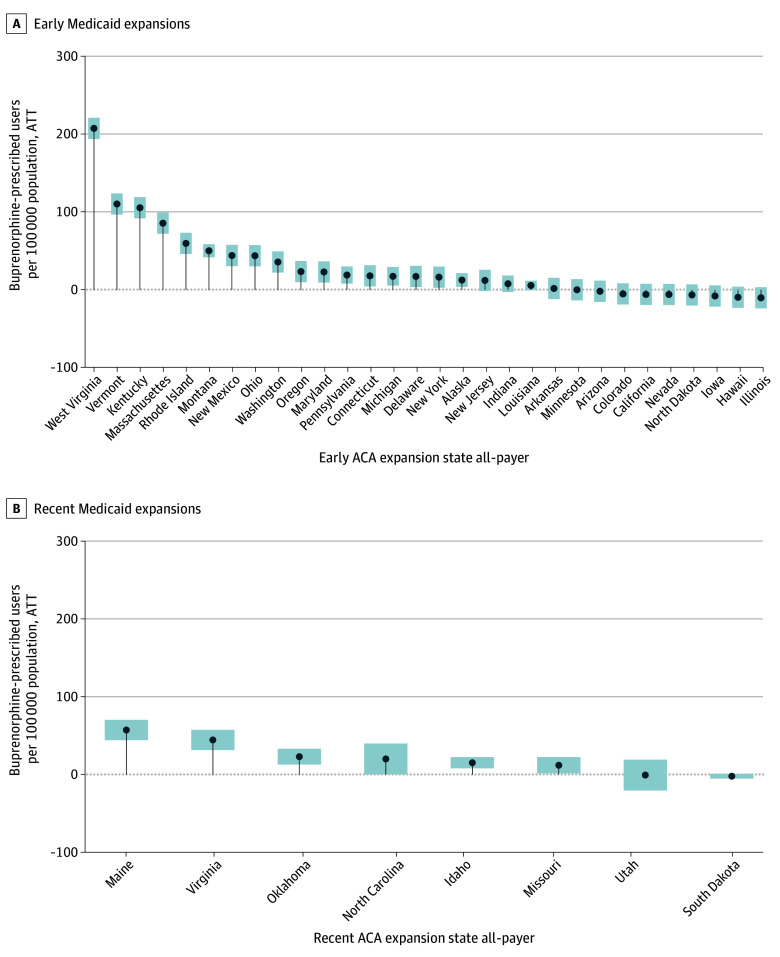
Difference-in-Differences Plot of State Case Studies: Medicaid Expansions and Buprenorphine Patients—All-Payer Average treatment effect on the treated (ATT) estimates using staggered adoption difference-in-differences estimation, covering 24 months before and 24 months after the start of each Medicaid expansion, was used to evaluate the average change in the rate of unique buprenorphine prescribed users per 100 000 of population in each state-month cell following the adoption of Medicaid for each treated state. Never-treated states were used as the control group. Data are from IQVIA longitudinal prescription database for 2013 to 2024, aggregating prescriptions for buprenorphine by state and month. Data for expansion dates from the Kaiser Family Foundation and state populations from US Census Bureau. The treatment group analysis is limited in Figure 2A to early adopters (expanded in 2014-2016) and Figure 2B to recent adopters (2019-2023; see eTable 1 in the Supplement). ACA indicates Patient Protection and Affordable Care Act.

The association between Medicaid expansion and buprenorphine treatment varied across states (Figure 2). Among recent-expansion states, Maine experienced the largest absolute increase (57.22 patients per 100 000; 95% CI, 44.20-70.24 per 100 000; 14.2% increase from baseline). Virginia showed the largest relative increase (64.24%), reflecting low baseline access of 69.2 per 100 000 (ATT, 44.46; 95% CI, 31.44-57.49), while Oklahoma also experienced substantial gains (22.98 per 100 000; 95% CI, 12.84-33.12 per 100 000), as well as Idaho (ATT, 20.04; 95% CI, 0.19-39.87). Other recently expanded states had smaller, generally positive or nonsignificant effects.

Within the early expansion cohort, high-burden states such as West Virginia, Vermont, and Kentucky experienced the largest postexpansion gains (West Virginia: 208 patients per 100 000; ATT, 207.58; 95% CI, 193.96-221.20; 49.3% increase from baseline; Vermont: 11 patients per 100 000; ATT, 110.37; 95% CI, 96.75-123.99; 34.4% increase; Kentucky: 105 patients per 100 000; ATT, 105.49 95% CI, 91.87-119.11; 33.6% increase from baseline), although ATTs in this cohort were modest and often not statistically significant (Figure 2A). Overall, outcomes associated with expansion were positive across most states, with larger gains in settings with greater baseline treatment gaps, particularly among recent-expansion, high-need states.

## Discussion

In this national study, Medicaid expansion was associated with substantial improvements in buprenorphine treatment for OUD, particularly in states that expanded in 2019 or later. Recent expansion states saw a significant 14.7% increase in the rate of patients receiving buprenorphine treatment per capita, whereas early expansion states (2014-2016) had smaller (approximately 7%), nonsignificant changes. The largest gains among recent adopters were observed in Maine, Virginia, and Oklahoma.

Notably and consistent with prior literature,^8,9^ the expansion-associated increases in buprenorphine treatment were concentrated among Medicaid-covered patients, indicating successful enrollment of previously uninsured or underinsured individuals. Importantly, we observed no significant reductions in treatment among those paying through commercial insurance, cash (uninsured), or Medicare in expansion vs nonexpansion states. Early expansions were associated with some increases in cash payments, possibly reflecting generally higher initial denial rates in Medicaid^13,14^ or spillover effects for utilization. Similarly, the small, nonsignificant increase in Medicare-paid treatment in expansion states could suggest positive externalities—for example, improved awareness and practitioner capacity might benefit all patients, not only Medicaid enrollees—but our data show these spillover changes were minimal.

Overall, our findings suggest that Medicaid expansion was associated with improved access to buprenorphine treatment for OUD, particularly in high-need populations. For policymakers, this suggests that expansion can meaningfully increase treatment uptake, but evaluations should account for shifts in payment sources when assessing a policy’s net benefits. Although expansion provides the financial foundation necessary to broaden OUD treatment access, its impact is amplified when paired with complementary state policies, such as streamlined prior authorization, telehealth support, and expanded prescribing authority, that facilitate MOUD delivery. These findings suggest that expansion is an essential but not standalone strategy, and coordinated policy efforts are needed to translate coverage gains into sustained treatment capacity. Our study contributes to the literature on Medicaid expansion and OUD treatment in several ways. First, we used post-2018 national data and examined the most recent wave of state expansions. This is important because the context of the opioid epidemic and the health care system has evolved since the initial expansions in 2014. States expanding after 2018 differed from earlier adopters with higher uninsurance rates and fewer OUD treatment resources. The states that remain without Medicaid expansions have even higher uninsurance,^15^ making our findings particularly relevant for those states still deliberating expansion. By analyzing data through 2024, our study provides an updated picture of how Medicaid expansion is associated with buprenorphine prescription dispensation trends over the past decade, incorporating the era of fentanyl-driven overdoses and the COVID-19 pandemic’s effects on substance use and telemedicine. In contrast to earlier studies that largely relied on data only up to 2017 to 2018 and traditional analytic methods,^7,8^ our approach provides more robust results by using a staggered-adoption DiD design that produces unbiased estimates even when treatment effects vary over time and across cohorts.^10^ We demonstrated that a naive 2-way fixed-effects model would have underestimated the outcomes of the recent expansions (essentially missing the outcome we found to be significant), underscoring the value of applying modern methods in policy evaluations with sequential rollouts.

Second, this study offers new payer-specific insights that have been underexplored in prior work. A key question for policymakers is whether Medicaid expansion brings more people into treatment (a pure expansion of access) or simply provides a new payment source for people who would have received treatment anyway (a substitution of payer, which is still important for reducing financial burden associated with commercial-insurance copayment or self-pay but has different population health implications). Prior research noted mixed or offsetting trends in buprenorphine prescriptions by payer. Some studies observed increases in Medicaid-funded treatment accompanied by decreases in uninsured or privately funded treatment^6,8,9^; those analyses often lacked a unified population denominator or robust methodology to quantify net effects. Crucially, by disaggregating outcomes by payer and standardizing rates, our study provides clearer results, reflecting an overall treatment expansion with access to affordable care and financial protection for patients and clinicians.

Our results have implications for state-level decisions and federal initiatives aimed at promoting equitable access to OUD treatment. States that have not yet expanded Medicaid can look to our findings for evidence that expansion may substantially increase treatment uptake among vulnerable populations with OUD, particularly in an environment that has become more conducive to treatment delivery. At the same time, stakeholders should note that measuring policy success purely by increases in Medicaid treatment counts could overstate the new treatments if some substitution from other payers occurs—hence a comprehensive evaluation should consider net treatment changes across all payers. Using a back-of-the-envelope calculation relying on assumptions from other literature, we estimate that 115 to 140 overdose deaths a year may have been averted due to Medicaid expansion (see eAppendix 2 in Supplement 1).

Our finding that recent Medicaid expansions were more associated with positive outcomes than earlier expansions warrant consideration of possible explanations. One such explanation is the difference in baseline conditions: recent-adopting states often had more residents lacking insurance and larger gaps in OUD treatment, so the expansion opened the door for a relatively larger influx of new treatment patients. Additionally, the expansions after 2018 coincided with a period of important policy and practice changes in OUD treatment nationally. The removal of the federal X-waiver requirement eliminated a significant barrier to buprenorphine prescribing, potentially increasing the pool of practitioners willing to treat OUD.^16,17^ Similarly, the rapid expansion of telehealth during 2020 to 2021 (accelerated by the COVID-19 pandemic) made it easier for patients to access buprenorphine treatment remotely, which may have particularly benefited rural areas and states with fewer clinicians.^18^ These changes applied nationwide, but states that expanded Medicaid more recently may have been better positioned to capitalize on them; when those states did expand, there were more prescribers available to meet the new demand. In addition, years of federal investment in MOUD delivery and the gradual expansion of the buprenorphine-waivered workforce (including the increase to the 275-patient limit) expanded treatment capacity by the late 2010s. As buprenorphine became more widely available and normalized as standard care, recent expanders likely benefited from a more mature treatment ecosystem, consistent with diffusion-of-innovation and normalization frameworks. In contrast, early expansions took place in 2014 when such infrastructure was more limited; some early expansion states faced practitioner capacity issues, which might have muted the immediate association of expansion with treatment rates. Another factor could be that the opioid overdose crisis intensified and evolved in the late 2010s (eg, rising fentanyl prevalence), potentially increasing the urgency of treatment needs in recent-expanding states.^1^ Policymakers and clinicians may have responded with greater efforts to link people to care once Medicaid coverage became available.

Our analysis cannot conclusively parse the contributions of these factors, but it underscores that the context of implementation matters—Medicaid expansion’s effectiveness can depend on parallel developments in the health care system and the burden of disease. Among early expansion states, the variability also appears regionally patterned: states in Appalachia and the industrial Midwest, areas with high opioid overdose burdens, showed larger postexpansion increases than Western or Plains states, a pattern consistent with prior work indicating that Medicaid expansion had the greatest impact in high-need regions with substantial treatment gaps.^19,20,21^

### Limitations

This study has several limitations. First, prescription fill data capture treatment receipt but not prescribing, adherence, retention, diagnoses, or clinical outcomes; thus, they serve only as a proxy for medication access. Second, the IQVIA LRx database omits some dispensing sources (eg, Veterans Health Administration, Indian Health Service, and certain opioid treatment programs), though these represent a small share of national buprenorphine use and are unlikely to vary systematically by expansion status.^22^ Relatedly, although IQVIA reports approximately 90% of retail pharmacy coverage, this estimate relies on proprietary aggregation methods and may vary across states and over time. Third, as an observational analysis, our DiD design relies on the parallel trends assumption; despite supportive event-study evidence, residual confounding from concurrent state policies cannot be excluded. Fourth, we did not isolate effects of other ACA provisions or national opioid initiatives. Fifth, payer-specific denominators are survey-derived and may introduce measurement error, likely affecting levels more than differences. Additionally, available data from 2013 through 2024 limit the preperiod for the early expansion states and recent follow-up, warranting continued evaluation as additional data become available.

## Conclusions

In this cross-sectional study, Medicaid expansion was associated with significant improvements in buprenorphine treatment access for opioid use disorder, especially in states that implemented expansion in recent years under evolving treatment landscapes. These results highlight Medicaid expansion as an effective policy lever to increase utilization of evidence-based OUD treatment. States that have not yet expanded Medicaid can expect that doing so may bring a considerable number of untreated individuals into care. As state and federal policymakers continue to pursue strategies to combat the opioid overdose epidemic, ensuring broad insurance coverage through Medicaid expansion (alongside measures that support increased clinician supply and harm-reduction efforts) may have synergistic effects on overdose reduction. Ultimately, improving treatment affordability in conjunction with supportive regulatory changes (such as telehealth and removal of prescribing restrictions), facilitate better outcomes in the ongoing fight against opioid-related morbidity and mortality.

## Notes

Supplement 1. eAppendix 1. Literature Review eAppendix 2. Description of the Staggered Adoption Difference-in-Differences Approach eTable 1. Timing of State ACA Expansions and Categories eTable 2. Medicaid Expansions and Buprenorphine Patients: All Payer, Coefficients of Pre-expansion Impacts eFigure 1. Medicaid Expansions and Buprenorphine Patients: All Payer, Not-Yet Treated Control eFigure 2. Recent Medicaid Expanders Alternative Staggered Treatment Methods: All Payer eFigure 3. Heterogeneity Analysis by Above or Below Median Opioid OD Rate: All Payer eFigure 4. Medicaid Expansions and Buprenorphine Patients: Medicaid Payer eFigure 5. Medicaid Expansions and Buprenorphine Patients: Commercial Payer eFigure 6. Medicaid Expansions and Buprenorphine Patients: Cash Payer eFigure 7. Medicaid Expansions and Buprenorphine Patients: Medicare Payer eFigure 8. Medicaid Expansions and Buprenorphine Patients: All Payer, Leave 1 Out Among Control States eFigure 9. Medicaid Expansions and Buprenorphine Patients: Falsification Test Among Control States eReferences.

Supplement 2. Data Sharing Statement
