# Supplementary material for: Medicaid Expansion and Buprenorphine Dispensing in Early vs Recent Expansion States
Source: JAMA Netw Open. 2026 Feb 18;9(2):e2559803. doi: 10.1001/jamanetworkopen.2025.59803 (PMC12917678; doi:10.1001/jamanetworkopen.2025.59803)
Supplement: Supplement 1. — eAppendix 1. Literature Review eAppendix 2. Description of the Staggered Adoption Difference-in-Differences Approach eTable 1. Timing of State ACA Expansions and Categories eTable 2. Medicaid Expansions and Buprenorphine Patients: All Payer, Coefficients of Pre-expansion Impacts eFigure 1. Medicaid Expansions and Buprenorphine Patients: All Payer, Not-Yet Treated Control eFigure 2. Recent Medicaid Expanders Alternative Staggered Treatment Methods: All Payer eFigure 3. Heterogeneity Analysis by Above or Below Median Opioid OD Rate: All Payer eFigure 4. Medicaid Expansions and Buprenorphine Patients: Medicaid Payer eFigure 5. Medicaid Expansions and Buprenorphine Patients: Commercial Payer eFigure 6. Medicaid Expansions and Buprenorphine Patients: Cash Payer eFigure 7. Medicaid Expansions and Buprenorphine Patients: Medicare Payer eFigure 8. Medicaid Expansions and Buprenorphine Patients: All Payer, Leave 1 Out Among Control States eFigure 9. Medicaid Expansions and Buprenorphine Patients: Falsification Test Among Control States eReferences. [file jamanetwopen-e2559803-s001.pdf]

Supplemental Online Content

Siegal N, Gupta S, Miles J, et al. Medicaid expansion and buprenorphine dispensing in early vs recent expansion states. *JAMA Netw Open*. 2026;9(2):e2559803.
doi:10.1001/jamanetworkopen.2025.59803

eAppendix 1. Literature Review
eAppendix 2. Description of the Staggered Adoption Difference-in-Differences Approach
eTable 1. Timing of State ACA Expansions and Categories
eTable 2. Medicaid Expansions and Buprenorphine Patients: All Payer, Coefficients of Pre-expansion Impacts
eFigure 1. Medicaid Expansions and Buprenorphine Patients: All Payer, Not-Yet Treated Control
eFigure 2. Recent Medicaid Expanders Alternative Staggered Treatment Methods: All Payer
eFigure 3. Heterogeneity Analysis by Above or Below Median Opioid OD Rate: All Payer
eFigure 4. Medicaid Expansions and Buprenorphine Patients: Medicaid Payer
eFigure 5. Medicaid Expansions and Buprenorphine Patients: Commercial Payer
eFigure 6. Medicaid Expansions and Buprenorphine Patients: Cash Payer
eFigure 7. Medicaid Expansions and Buprenorphine Patients: Medicare Payer
eFigure 8. Medicaid Expansions and Buprenorphine Patients: All Payer, Leave 1 Out Among Control States
eFigure 9. Medicaid Expansions and Buprenorphine Patients: Falsification Test Among Control States
eReferences.

This supplemental material has been provided by the authors to give readers additional information about their work.

© 2026 Siegal N et al. *JAMA Network Open*.

eAppendix 1. Literature Review

Prior studies, mainly focused on the early-expansion states, evaluated the effects of the Affordable Care Act (ACA) Medicaid expansions on access to OUD medication, with particular attention to prescribing trends for buprenorphine, but also some studies on methadone.[1–11] Using national or multi-state pharmacy claims data, prior studies generally find that Medicaid expansion increased Medicaid-financed buprenorphine prescribing, though evidence on net treatment gains and payer substitution remains mixed.[1–3] Some studies looked at substance use treatment admissions using the Treatment Episode Data Set (TEDS).[5–8] Most of these studies, however, focus on early expansion years and do not use contemporary DID econometric approaches that account for policy variation across staggered adopters, or use very recent data. Thus, our study fills multiple gaps in the literature.

Golan et al. (2023) analyze buprenorphine treatment from 2009 to 2018, examining heterogeneity in expansion effects by county rurality and income. The authors find that Medicaid expansion reduced income-related differences in buprenorphine use in urban counties but not in rural areas.[1] However, the study does not disaggregate results by payer or take advantage of testing assumptions related to causal inference methods. Like much of the literature, it uses a traditional pre/post design and does not address treatment effect heterogeneity or differential timing of state expansion decisions.

Knudson, Hartman, and Walsh (2022) also use IQVIA data from 2013–2018, to study buprenorphine utilization, reporting increases in Medicaid-paid and total prescriptions in expansion states, but point estimates were statistically insignificant for these results, although they do find significant decreases in cash pay, Medicare pay, and commercial prescriptions.[2] This suggests potential substitution across payers. While the authors attempted to assess variation in expansion timing, they note that staggered DID produced estimation challenges in their context. Similarly, Olfson et al. (2021) use IQVIA data through 2018 and document increased Medicaid-paid buprenorphine prescriptions following expansion but find that overall treatment increases in expansion and non-expansion states were similar due to offsetting trends among other payers.[3] Saloner et al. (2018) examine five states from 2010 to 2015 (also using

© 2026 Siegal N et al. *JAMA Network Open*.

IQVIA) and find significant increases in buprenorphine and naloxone prescriptions per capita in expansion counties, although without strong overall effects.[12]

Some studies used only Medicaid data, most often the State Drug Utilization Data (SDUD). Wen et al., (2017) used 2010-2017 SDUD data to show that in states that expanded Medicaid in 2014, Medicaid-covered buprenorphine prescriptions rose nearly 70% and spending increased roughly 50% compared to states without early expansion.[11] One paper used federal data from the Automation of Reports and Consolidated Orders System (ARCOS), which tracks the distribution of controlled substances, and the Centers for Disease Control and Prevention (CDC). Shakya and Harris (2022) used state-level data on retail opioid prescriptions from the CDC and buprenorphine distributions from ARCOS between 2006 and 2017 in a DID framework to examine whether Medicaid expansion combined with pain management "pill mill" laws influenced buprenorphine use. They found that Medicaid expansion was associated with increased buprenorphine availability primarily in states that also implemented stringent supply-side regulations targeting inappropriate opioid prescribing, suggesting complementary effects of insurance coverage and opioid-control policies.[13] One study used national-level Medicaid data which, like SDUD, does not include data from other payers. Sharp et al. (2018), using Medicaid claims data from 2011–2016, report over a 200% increase in buprenorphine and naltrexone prescribing in expansion states.[9] Swartz et al., (2023) use TEDS admissions data covering the years 2006 through 2019 to show expansion states experienced increased Medicaid enrollment and MOUD-inclusive treatment plans for both housed and homeless individuals.[10]

Other studies look at medications for OUD (MOUD) from treatment programs. Abraham et al. (2021) found that Medicaid expansion was associated with significant increases in the availability of MOUD, particularly buprenorphine and injectable naltrexone, among opioid treatment programs (OTPs), especially nonprofit facilities. However, they observed no consistent changes in non-OTP specialty treatment programs, which make up the majority of the treatment system.[4] These findings suggest that while Medicaid expansion improved access within a narrow subset of providers, its broader impact on the specialty treatment system was limited.

A set of papers look at treatment admissions using the Treatment Episode Data Set (TEDS) which captures some OTPs but also intensive inpatient (as well as outpatient). Choi et al. (2021)

© 2026 Siegal N et al. *JAMA Network Open*.

examined Medicaid expansion's effects on initiation of MOUD among pregnant women, using TEDS discharge data from 2010–2018. Their analysis finds that states without punitive statutes prohibiting substance use during pregnancy saw a 15.3 percentage-point increase in MOUD initiation two years post-expansion—compared with only a 1.5 percentage-point increase in states with such prohibitions—demonstrating that criminalizing prenatal substance use may significantly dampen Medicaid expansion's positive impact on treatment access.[5] Datta, Oglesby, and George (2022) also used TEDS admissions data to show that Medicaid expansion was associated with large increases, ranging from 20 to 33 percentage points, in OUD treatment admissions among Medicaid beneficiaries in expansion states, particularly among newly eligible adults aged 30–34 and older adults over 55.[6] Khatri, Howell, and Winkelman (2021) analyzed TEDS admissions data too, from 2008 to 2017 and found that among individuals referred for care by criminal justice agencies, receipt of MOUD increased by 165% in Medicaid expansion states compared to non-expansion states.[7] Despite this improvement, justice-involved individuals remained significantly less likely than those referred through other channels to receive MOUD, indicating that expansion reduced but did not eliminate gaps. Meinhofer and Witman (2018), using TEDS admissions data from 2007 to 2016, found a 113% increase in Medicaid-paid OUD admissions in expansion states, without crowding out admissions covered by other payers.[8] A recent trends analysis shows that post-2018 Medicaid expansions were followed by increased Medicaid-paid and all-payer buprenorphine use; these suggestive but descriptive findings indicate the need for closer examination of the relationship between recent Medicaid expansion and increased buprenorphine use (cite HA paper).

In contrast to these earlier studies, our applies modern difference-in-differences methods that address recent critiques of traditional approaches. Specifically, we implement the estimator proposed by Callaway and Sant'Anna, which accommodates staggered policy adoption and allows for treatment effect heterogeneity across expansion cohorts. Our study also separates effects by payer, a crucial part of showing if substitution occurs and impacts on total treatment use. We show Medicaid, Medicare, commercial, and cash pay, providing a more detailed understanding of how Medicaid expansion affected buprenorphine prescribing patterns. In doing so, we extend prior research to a more recent period, incorporate a broader set of expansion

© 2026 Siegal N et al. *JAMA Network Open*.

states, and apply more robust empirical methods to isolate causal effects, showing differences for earlier and recent expanders.

© 2026 Siegal N et al. *JAMA Network Open*.

eAppendix 2. Description of the Staggered Adoption Difference-in-Differences Approach

Statistical Estimation Details: We employed the Callaway and Sant'Anna (2021)[14]difference-in-differences estimator to handle multiple treatment adoption times. In this framework, we estimate group-time average treatment effects on the treated (ATT) for each cohort *g* (defined by the state's expansion year and month) at each period *t* relative to that cohort's expansion implementation. Formally, our main model can be represented as:

$$Y_{s,t} = \alpha_s + \lambda_t + \sum_{\tau \geq 0}^{g \in G} \delta_{g,\tau} \cdot 1\{G_s = g\} \cdot 1\{t - T_g = \tau\} + \varepsilon_{s,t},$$

where $Y_{s,t}$ is the outcome (e.g., all-payer buprenorphine treatment rate) in state *s* at time *t*; α_s are state fixed effects; λ_t are time (month-year) fixed effects; G_s denotes the expansion cohort of state *s* (defined by the implementation year of expansion, or "Never" for non-expansion states); T_g is the calendar time of cohort *g*'s expansion; and $\tau = t - T_g$ indexes the time relative to expansion. The coefficient $\delta_{g,\tau}$ represents the effect for cohort *g* in relative period τ (with $\tau = 0$ as the first post-expansion month, and negative values for leads/pre-period). The summation term thus captures cohort- and timing-specific impacts. All models include year–month fixed effects, which account for nationwide secular trends in buprenorphine utilization—such as evolving clinical guidelines, federal regulatory changes, or shifts in overdose risk—that affect all states. By comparing expansion states to contemporaneous non-expansion states, the design isolates the relative effect of Medicaid expansion from these broader trends.

The average treatment effect on the treated (ATT) for a given cohort *g* in the post-expansion period can be obtained by aggregating the $\delta_{g,\tau}$ for $\tau \geq 0$ (or at specific post-periods of interest). In practice, we report the overall post-expansion ATT for early adopters and for recent adopters, which are weighted averages of the cohort- and time-specific effects for each group. Non-expanding states (never-treated) serve as the comparison group to estimate these effects, and the estimator uses an appropriate weighting scheme (based on cohort and time proportions) as described in Callaway & Sant'Anna (2021). All models were estimated using the "csdid" package in Stata, which implements this two-stage procedure to first compute group-time ATTs and then aggregate them.

A difference-in-differences framework was used because Medicaid expansions occurred at different times across states and staggered-adoption DiD estimators provide unbiased estimates under treatment-effect heterogeneity. We verified that our results are robust to using alternative implementations (e.g., a stacked regression approach with cohort-specific indicators, and a interaction-weighted estimator), which produced very similar estimates (see eFigure B2 for a comparison including a traditional two-way fixed-effects model).

Parallel Trends Assessment: The identification assumption in DiD is that, in the absence of treatment, the outcome trajectories of treated and control units would have followed parallel paths. We assessed this assumption by conducting an event-study analysis for each outcome. This involved estimating the coefficients $\delta_{g,\tau}$ for a series of leads (pre-expansion periods) and lags (post-expansion periods) for each expansion cohort and plotting them relative to the expansion date (with an appropriate reference period before expansion). We present these event-study plots in Figure 1 (all-payer outcome) and in eFigures A4-A7 (payer-specific outcomes).

© 2026 Siegal N et al. *JAMA Network Open*.

The figures indicate that there were no significant pre-expansion differences between expansion and control states – the lead coefficients around $\tau = -1$ (one year before expansion) are close to zero and not statistically significant for all outcomes, supporting the validity of the parallel trends assumption. Further, eTable 2 presents the pre-expansion coefficients and significance levels to confirm the lack of pre-trends. Additionally, the event-study plots show the dynamic effects of expansion over time: for example, the increase in Medicaid-paid buprenorphine prescribing occurs rapidly within the first few months post-expansion and is sustained, whereas the decline in commercial-paid prescribing in recent expansion states emerges more gradually (eFigure B5). These dynamic patterns provide insight into how quickly expansion effects materialize.

Alternative Control Group Specification: In our main analysis, we treated the 10 states that never expanded Medicaid (through 2024) as the control group for all expansion cohorts. As a robustness check, we re-estimated the effects using a "not-yet-treated" approach: in this specification, states that eventually expanded (recent adopters) could serve as controls for early adopters in the period before their own expansion took effect. This approach can increase the effective control sample size for early expansion effects and is another way to mitigate biases from time-varying unobservables. The not-yet-treated estimator is also implemented in the Callaway & Sant'Anna framework by choosing an appropriate reference group (we used the built-in option to use "not yet treated" as controls). The results (presented in eFigure B1) were nearly identical to those from the main specification. In fact, because the majority of expansions occurred in 2014 and only a handful expanded later, the distinction between using never-treated vs not-yet-treated was minimal for the early cohort's estimation.

Subgroup and Sample Restriction Analyses: Although our primary stratification of interest was by expansion timing (early vs recent adopters), we conducted additional subgroup analyses similar to those often examined in Medicaid expansion studies to see if certain populations experienced different effects. We stratified our data by state baseline OUD severity (as mentioned, high vs low overdose mortality states; results showed no significant interaction). Because individual-level demographic subgroups (such as race or age) could not be directly analyzed with state-level aggregate data, we could not assess heterogeneity in expansion effects by patient demographics in this study; that would require microdata or survey data linkage, which is outside our scope.

Additional Figures and Tables: In the Supplement, eTable 1 lists each expansion state and the date of Medicaid expansion implementation. eTable 2 presents the pre-expansion coefficients and statistics to further show the lack of pre-trends in both study periods. Figure 1 depicts the event-study plot for all-payer buprenorphine treatment rates, showing the parallel pre-trends and the post-expansion effect trajectories for early and recent cohorts. eFigure B2 includes a comparison of different DiD estimators (our preferred method vs a standard two-way fixed effects model), illustrating how the latter underestimates the recent-expansion effect. eFigures A4–A7 show event-study plots for each payer-specific outcome (Medicaid, commercial, cash, Medicare respectively), for early and recent cohorts. Figure 2 maps the state-specific all-payer ATT estimates, highlighting geographic variation in expansion effects.

© 2026 Siegal N et al. *JAMA Network Open*.

Overall, the supplemental analyses support the main conclusion that Medicaid expansion – especially the expansions occurring after 2018 – has led to meaningful increases in buprenorphine treatment engagement at the population level, without evidence of violating key assumptions or being driven by extraneous factors.

© 2026 Siegal N et al. *JAMA Network Open*.

eTable 1. Timing of State ACA Expansions and Categories

Notes: Authors presentation of timing of Medicaid expansion dates for each state from Kaiser Family Foundation's (KFF) Status of State Medicaid Expansion Decisions. Original 25 states to adopt the ACA expansion on January 1st, 2014 and 12 additional states to adopt through 2016 are categorized as "early adopters," while the 9 states to expand after 2018 are categorized as "recent adopters."
Source (KFF: https://www.kff.org/status-of-state-medicaid-expansion-decisions/)

Initial ACA Adopters (1/1/2014)	Arizona, Arkansas, California, Colorado, Connecticut, Delaware, District of Columbia, Hawaii, Illinois, Iowa, Kentucky, Maryland, Massachusetts, Minnesota, Nevada, New Jersey, New Mexico, New York, North Dakota, Ohio, Oregon, Rhode Island, Vermont, Washington, West Virginia	
Other Early Adopters (after 1/1/2014-2016)	**State**	**Adoption date**
	Alaska	9/1/15
	Indiana	2/1/15
	Louisiana	7/1/16
	Michigan	4/1/14
	Montana	1/1/16
	New Hampshire	8/15/14
	Pennsylvania	1/1/15
	Alaska	9/1/15
	Indiana	2/1/15
	Louisiana	7/1/16
	Michigan	4/1/14
	Montana	1/1/16
Recent Adopters (2019-2023)	**State**	**Adoption date**
	Idaho	1/1/20
	Maine	1/10/19
	Missouri	10/1/21
	Nebraska	10/1/20
	North Carolina	12/1/23
	Oklahoma	7/1/21
	South Dakota	7/1/23
	Utah	1/1/20
	Virginia	1/1/19

© 2026 Siegal N et al. *JAMA Network Open*.

eTable 2. Medicaid Expansions and Buprenorphine Patients: All Payer, Coefficients of Pre-expansion Impacts

Note: A staggered adoption difference-in-differences event study plot, covering 24 months before the start of each Medicaid expansion, was used to evaluate the rate of buprenorphine prescribed users per 100,000 of population in each state-month cell. Never treated states were included in the control group. Data are from IQVIA LRx for 2013-2024, aggregating prescriptions for buprenorphine by state and month. Data for expansion dates from KFF and state populations from US Census Bureau. We limit the treatment group analysis in Column A to early adopters (expanded in 2014-2016) Column B to recent adopters (2019-2023, see Supplement eTable B1).

**** p<0.001, ** p<0.01, *p<0.05*

	A. Early Expansions				B. Recent Expansions		
**Event Time**	**Coefficient**	**p-Value**	**95% CI**		**Coefficient**	**p-Value**	**95% C**
-24	-3.608	0.604	-17.237	10.020	0.541	0.473	-0.937
-23	-0.286	0.784	-2.335	1.762	0.795	0.256	-0.576
-22	-0.897	0.277	-2.514	0.720	5.365**	0.007	1.466
-21	-0.744	0.740	-5.137	3.647	-1.866	0.446	-6.674
-20	-11.397	0.514	-45.661	22.867	1.084	0.195	-0.556
-19	-10.727	0.448	-38.467	17.011	0.750	0.403	-1.008
-18	2.053	0.881	-24.880	28.987	7.348	0.156	-2.815
-17	13.650	0.358	-15.444	42.745	4.65*	0.018	0.800
-16	2.976	0.644	-9.666	15.619	1.876	0.311	-1.754
-15	1.750	0.268	-1.345	4.846	0.532	0.561	-1.264
-14	-0.754	0.522	-3.063	1.554	-0.090	0.868	-1.159
-13	6.833	0.277	-5.484	19.152	-1.414	0.337	-4.300
-12	0.588	0.628	-1.794	2.971	-3.912	0.243	-10.481
-11	-1.880	0.136	-4.351	0.589	1.174	0.621	-3.479
-10	-1.201	0.384	-3.904	1.502	0.746	0.430	-1.109
-9	-2.164	0.014	-3.885	-0.445	-0.710	0.756	-5.199
-8	0.272	0.747	-1.382	1.927	-4.097	0.063	-8.414
-7	-4.771	0.428	-16.557	7.015	2.688	0.350	-2.950
-6	0.318	0.893	-4.325	4.961	1.631	0.251	-1.152
-5	3.338	0.452	-5.363	12.040	-1.283	0.664	-7.067
-4	1.320	0.710	-5.631	8.271	0.001	0.999	-3.327
-3	0.146	0.844	-1.315	1.608	6.177*	0.014	1.233
-2	-0.173	0.703	-1.065	0.718	-5.631	0.268	-15.604
-1	-3.608	0.604	-17.237	10.020	0.541	0.473	-0.937

© 2026 Siegal N et al. *JAMA Network Open*.

eFigure 1. Medicaid Expansions and Buprenorphine Patients: All Payer, Not-Yet Treated Control

Note: A staggered adoption difference-in-differences event study plot, covering 24 months before and 24 months after the start of each Medicaid expansion, was used to evaluate the rate of buprenorphine prescribed users per 100,000 of population in each state-month cell. Never- and not-yet treated states were included in the control group. Data are from IQVIA LRx for 2013-2024, aggregating prescriptions for buprenorphine by state and month. Data for expansion dates from KFF and state populations from US Census Bureau. We limit the treatment group analysis in eFigure B1a to early adopters (expanded in 2014-2016) and eFigure B1b to recent adopters (2019-2023, see Supplement eTable B1).

eFigure 1a: Early Medicaid Expansions

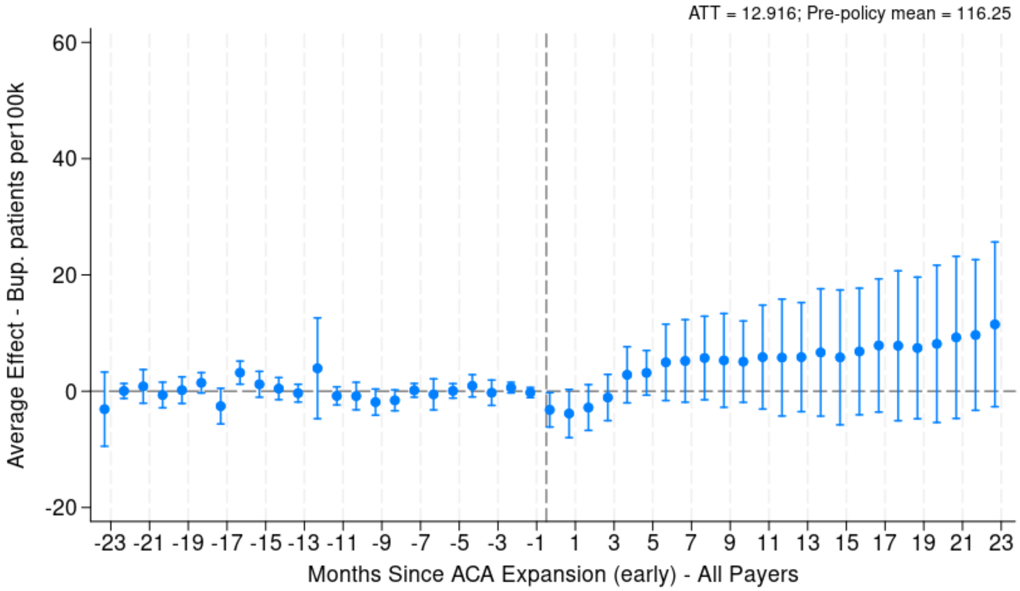

eFigure 1b: Recent Medicaid Expansions

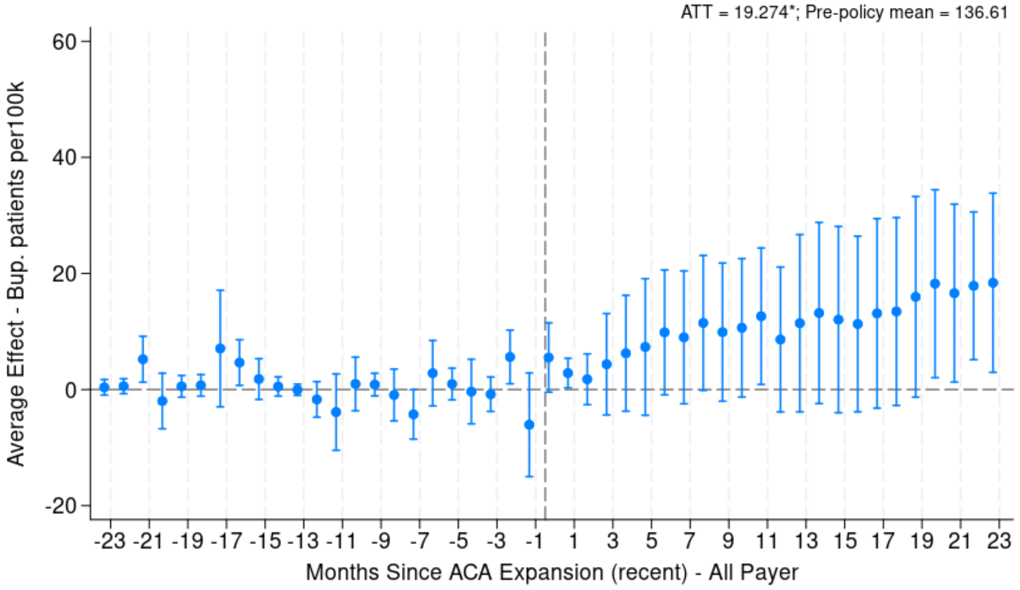

© 2026 Siegal N et al. *JAMA Network Open*.

eFigure 2. Recent Medicaid Expanders Alternative Staggered Treatment Methods: All Payer

Note: Average treatment effect on the treated (ATT) of various difference-in-differences event study models, from left to right: standard two-way fixed-effects regressions, followed by three models which adjust for the heterogeneity introduced due to the staggered timing of policy adoption: Callaway & Sant'Anna[14], Sun & Abraham,[15] and Gardner et. al.[16] methods. Each point, covering 24 months before and 24 months after the start of each Medicaid expansion, was used to evaluate the average change in the rate of unique buprenorphine prescribed users per 100,000 of population in each state-month cell following the adoption of Medicaid expansion. Never- treated states were used as the control group. Data are from IQVIA LRx for 2013-2024, aggregating prescriptions for buprenorphine by state and month. Data for expansion dates from KFF and state populations from US Census Bureau. We limit the treatment analysis to recent adopters (2019-2023, see Supplement eTable B1).

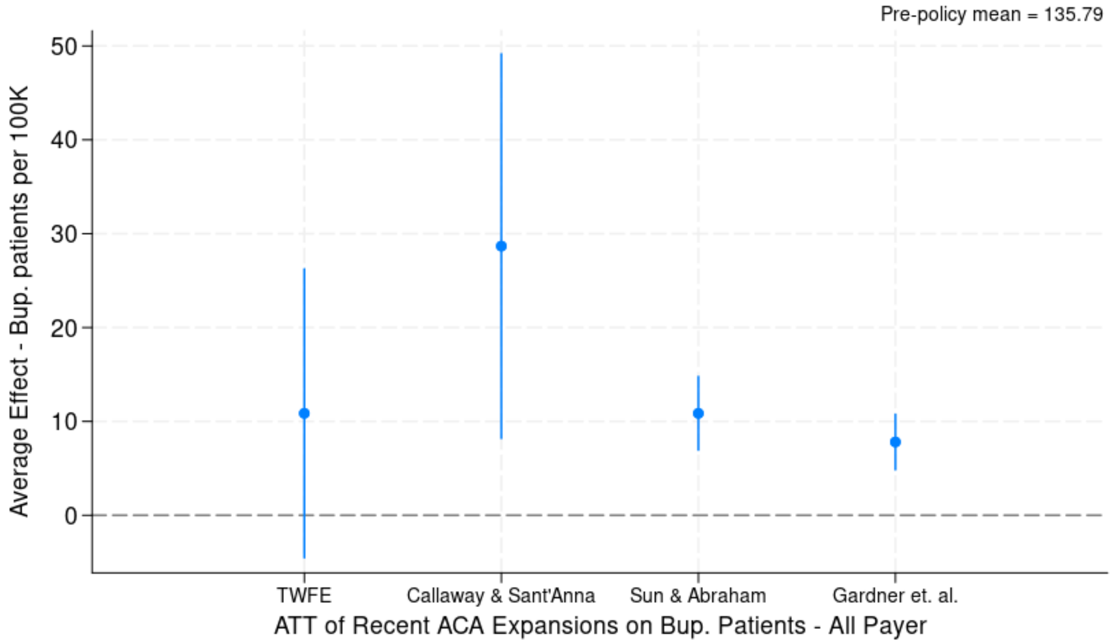

© 2026 Siegal N et al. *JAMA Network Open*.

eFigure 3. Heterogeneity Analysis by Above or Below Median Opioid OD Rate: All Payer

Note: Average treatment effect on the treated (ATT) estimates using staggered adoption difference-in-differences estimation, covering 24 months before and 24 months after the start of each Medicaid expansion, was used to evaluate the average change in the rate of unique buprenorphine prescribed users per 100,000 of population in each state-month cell following the adoption of Medicaid expansion among pre-period (2012) above or below median opioid overdose mortality rate states. Never-treated states were used as the control group. Data are from IQVIA LRx for 2013-2024, aggregating prescriptions for buprenorphine by state and month. Data for expansion dates from KFF and state populations from US Census Bureau. We limit the treatment group analysis in eFigure B3a to early adopters (expanded in 2014-2016) and eFigure B3b to recent adopters (2019-2023, see Supplement eTable B1).

eFigure 3a: Early Medicaid Expansions

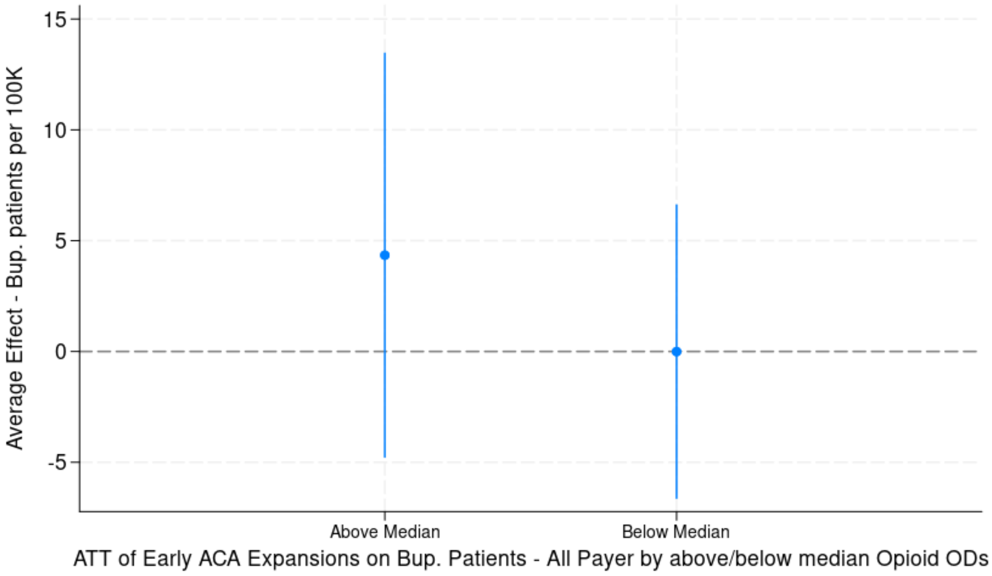

eFigure 3b: Recent Medicaid Expansions

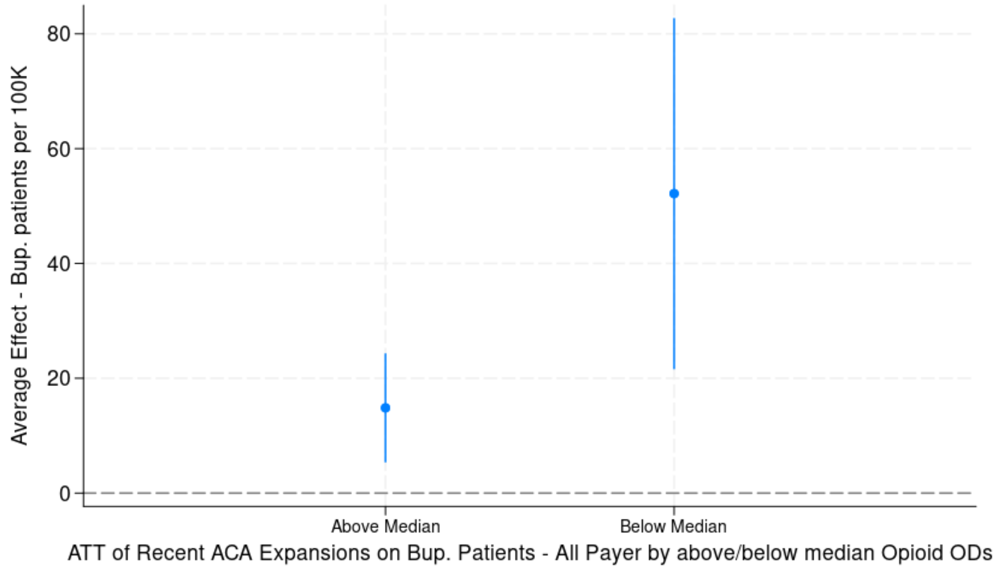

© 2026 Siegal N et al. *JAMA Network Open*.

eFigure 4. Medicaid Expansions and Buprenorphine Patients: Medicaid Payer

Note: A staggered adoption difference-in-differences event study plot, covering 24 months before and 24 months after the start of each Medicaid expansion, was used to evaluate the rate of Medicaid-paid buprenorphine prescribed users per 100,000 Medicaid enrollees in each state-month cell. Never-treated states were used as the control group. Data are from IQVIA LRx for 2013-2024, aggregating prescriptions for buprenorphine by payer, state, and month. Data for expansion dates and enrollee population from KFF. We limit the treatment group analysis in eFigure B6a to early adopters (expanded in 2014-2016) and eFigure B6b to recent adopters (2019-2023, see Supplement eTable B1).

eFigure 4a: Early Medicaid Expansions

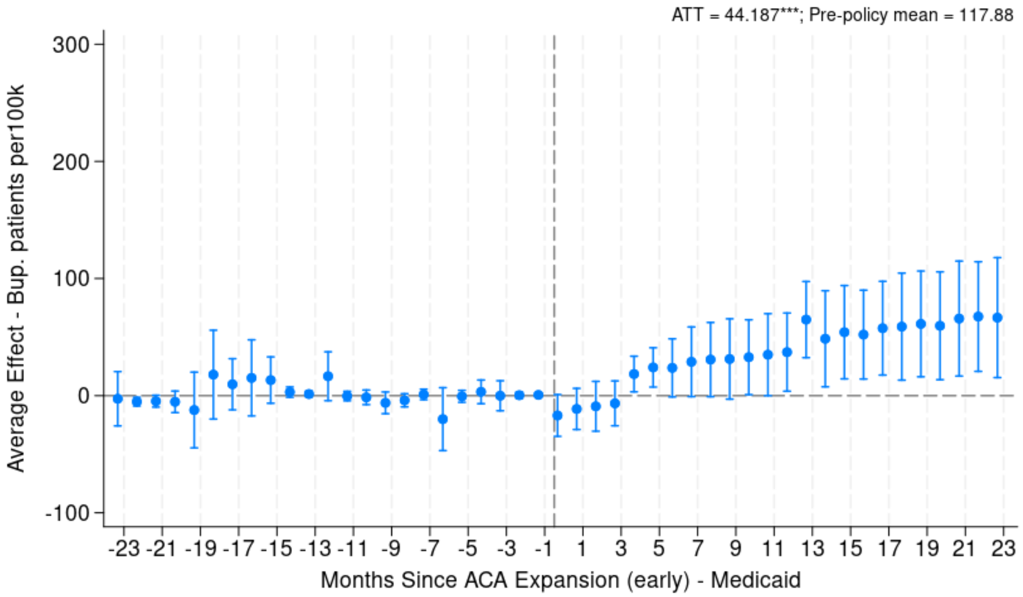

eFigure 4b: Recent Medicaid Expansions

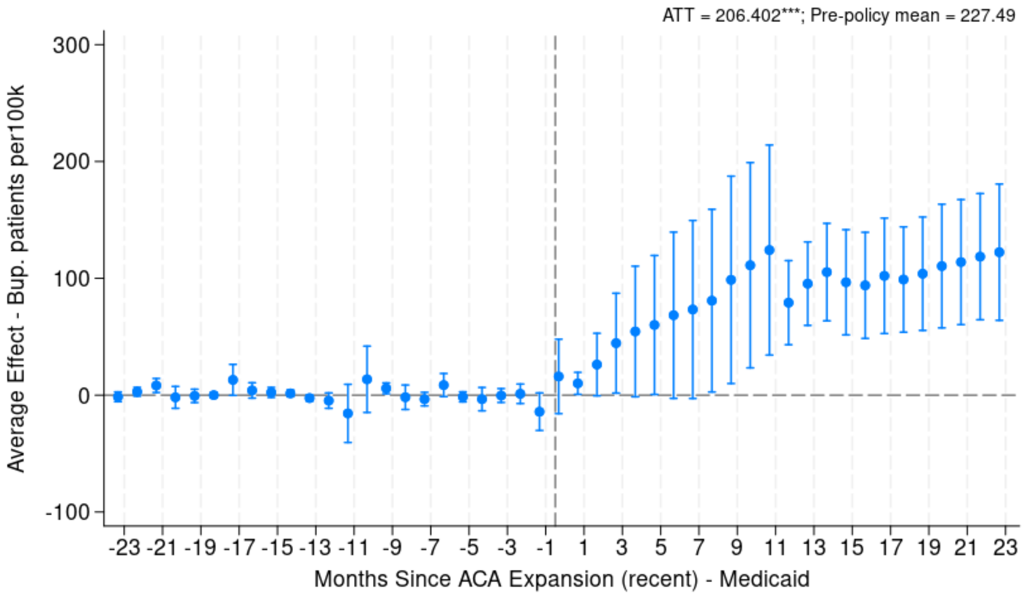

© 2026 Siegal N et al. *JAMA Network Open*.

eFigure 5. Medicaid Expansions and Buprenorphine Patients: Commercial Payer

Note: A staggered adoption difference-in-differences event study plot, covering 24 months before and 24 months after the start of each Medicaid expansion, was used to evaluate the rate of commercial-paid buprenorphine prescribed users per 100,000 privately insured population in each state-month cell. Never-treated states were used as the control group. Data are from IQVIA LRx for 2013-2024, aggregating prescriptions for buprenorphine by payer, state, and month. Data for expansion dates and insurance population from KFF. We limit the treatment group analysis in eFigure B5a to early adopters (expanded in 2014-2016) and eFigure B5b to recent adopters (2019-2023, see Supplement eTable B1).

eFigure 5a: Early Medicaid Expansions

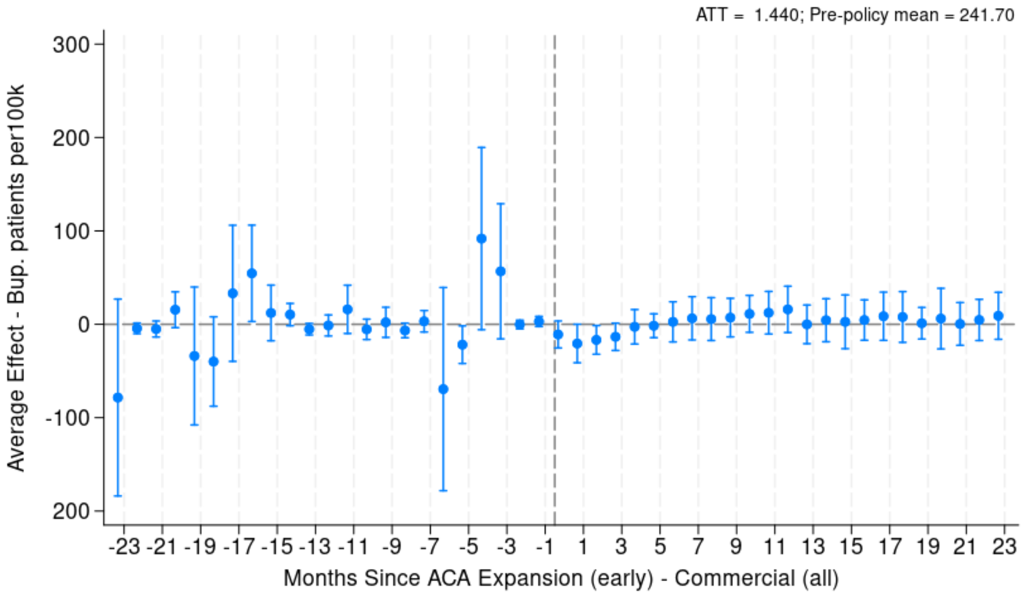

eFigure 5b: Recent Medicaid Expansions

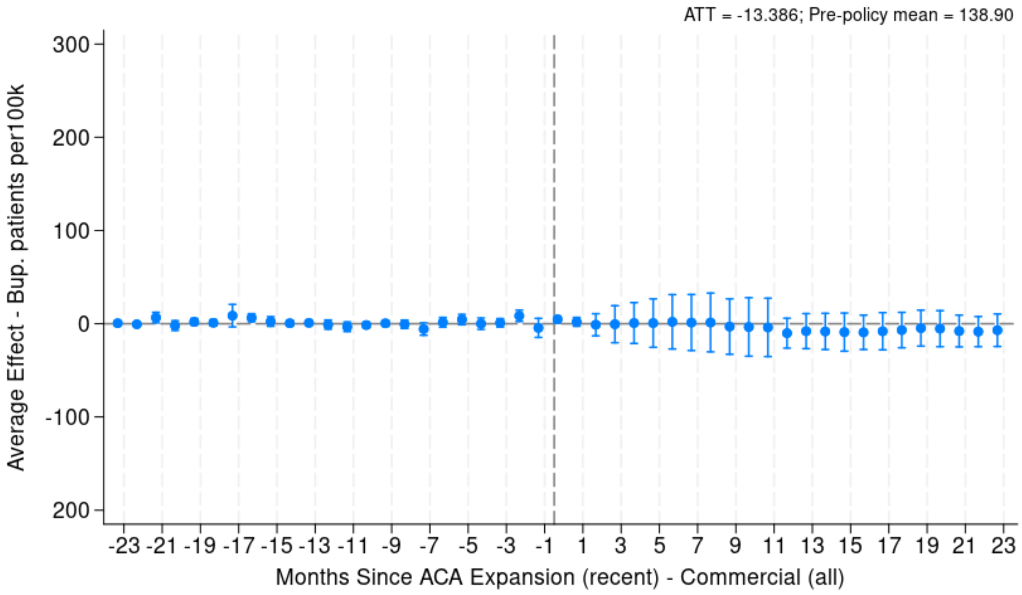

© 2026 Siegal N et al. *JAMA Network Open*.

eFigure 6. Medicaid Expansions and Buprenorphine Patients: Cash Payer

Note: A staggered adoption difference-in-differences event study plot, covering 24 months before and 24 months after the start of each Medicaid expansion, was used to evaluate the rate of cash (self)-paid buprenorphine prescribed users per 100,000 uninsured population in each state-month cell. Never-treated states were used as the control group. Data are from IQVIA LRx for 2013-2024, aggregating prescriptions for buprenorphine by payer, state, and month. Data for expansion dates and uninsured population from KFF. We limit the treatment group analysis in eFigure B6a to early adopters (expanded in 2014-2016) and eFigure B6b to recent adopters (2019-2023, see Supplement eTable B1).

eFigure 6a: Early Medicaid Expansions

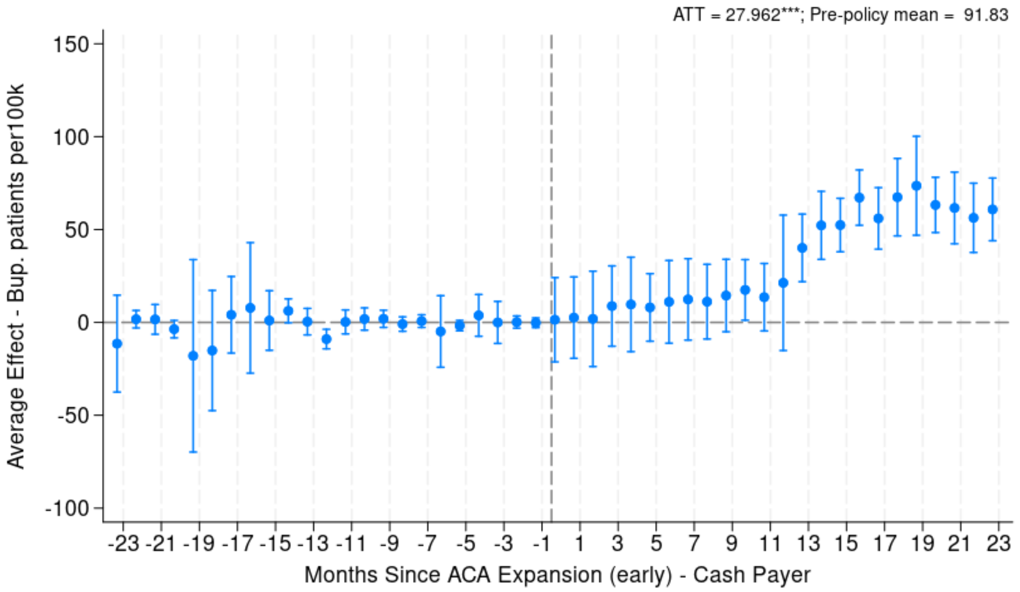

eFigure 6b: Recent Medicaid Expansions

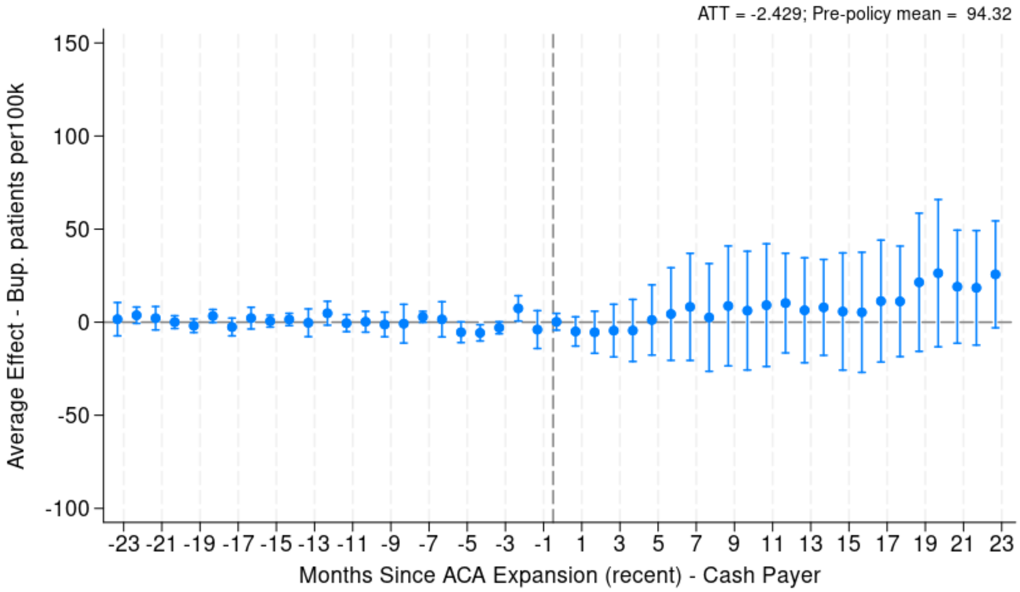

© 2026 Siegal N et al. *JAMA Network Open*.

eFigure 7. Medicaid Expansions and Buprenorphine Patients: Medicare Payer

Note: A staggered adoption difference-in-differences event study plot, covering 24 months before and 24 months after the start of each Medicaid expansion, was used to evaluate the rate of Medicare-paid buprenorphine prescribed users per 100,000 Medicare enrollees in each state-month cell. Never-treated states were used as the control group. Data are from IQVIA LRx for 2013-2024, aggregating prescriptions for buprenorphine by payer, state, and month. Data for expansion dates and Medicare enrollees from KFF. We limit the treatment group analysis in eFigure B7a to early adopters (expanded in 2014-2016) and eFigure B7b to recent adopters (2019-2023, see Supplement eTable B1).

eFigure 7a: Early Medicaid Expansions

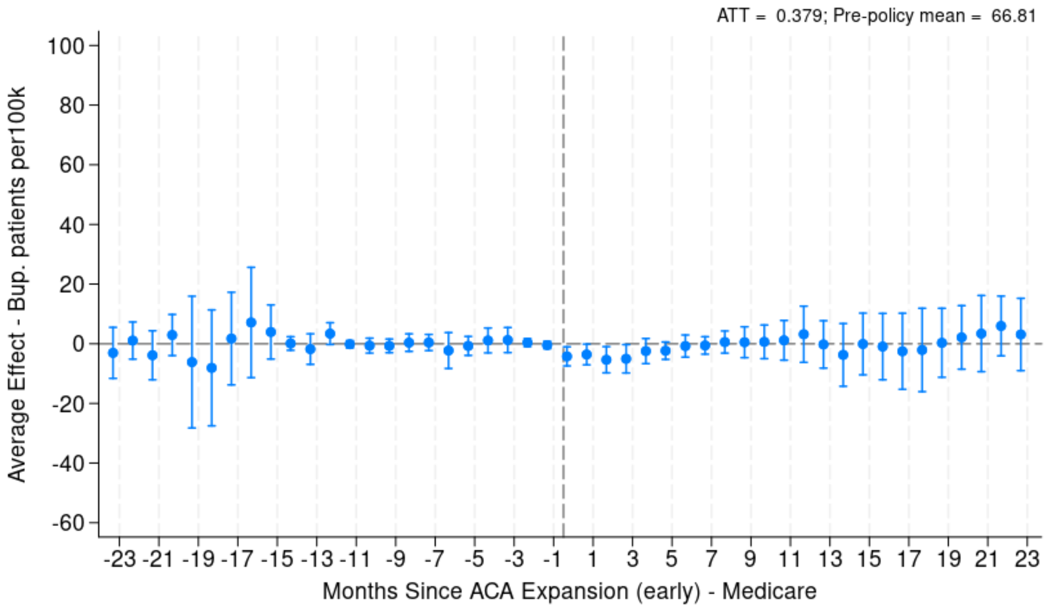

eFigure 7b: Recent Medicaid Expansions

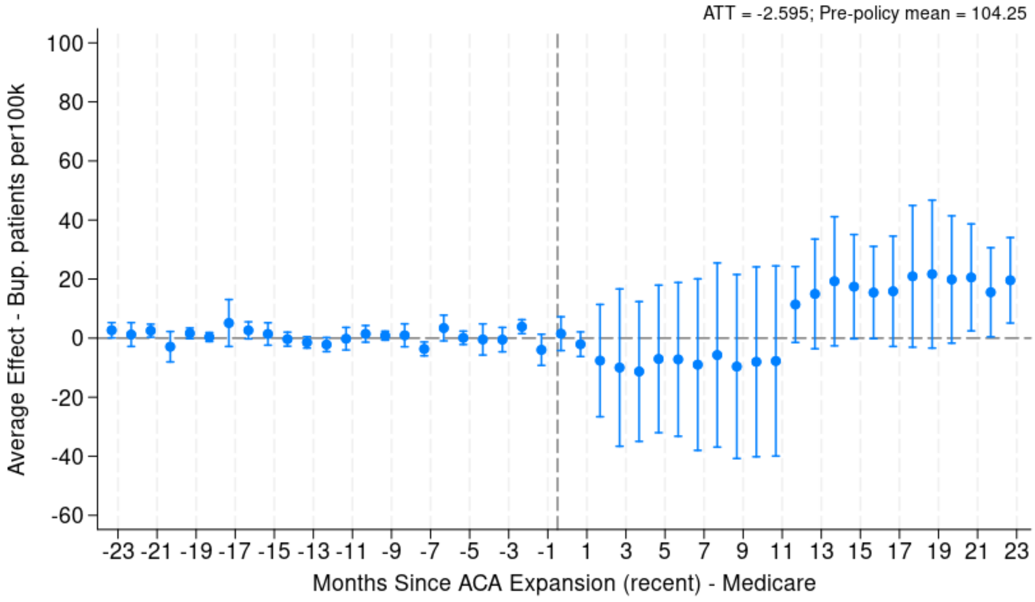

© 2026 Siegal N et al. *JAMA Network Open*.

eFigure 8. Medicaid Expansions and Buprenorphine Patients: All Payer, Leave 1 Out Among Control States

Note: A plot of average treatment effects on the treated from staggered adoption difference-in-difference regressions, covering 24 months before and 24 months after the start of each Medicaid expansion, used to evaluate the rate of buprenorphine prescribed users per 100,000 of population in each state-month cell. Never-treated states were included in the control group, with one control state excluded from each specification as noted below on the x axis. Data are from IQVIA LRx for 2013-2024, aggregating prescriptions for buprenorphine by state and month. Data for expansion dates from KFF and state populations from US Census Bureau. We limit the treatment group analysis in eFigure B8a to early adopters (expanded in 2014-2016) and eFigure B8b to recent adopters (2019-2023, see Supplement eTable B1).

eFigure 8a: Early Medicaid Expansions

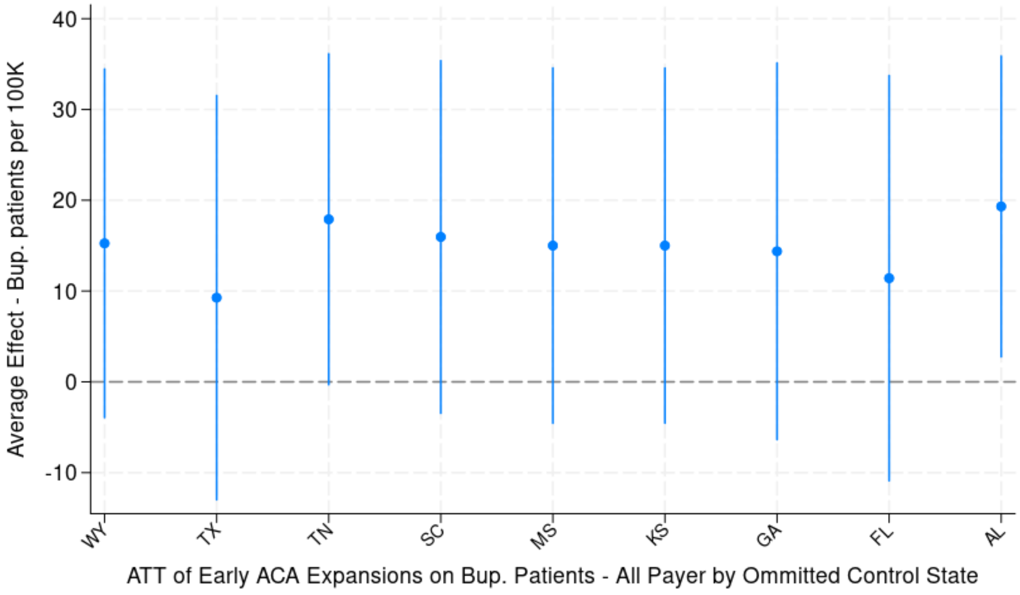

eFigure 8b: Recent Medicaid Expansions

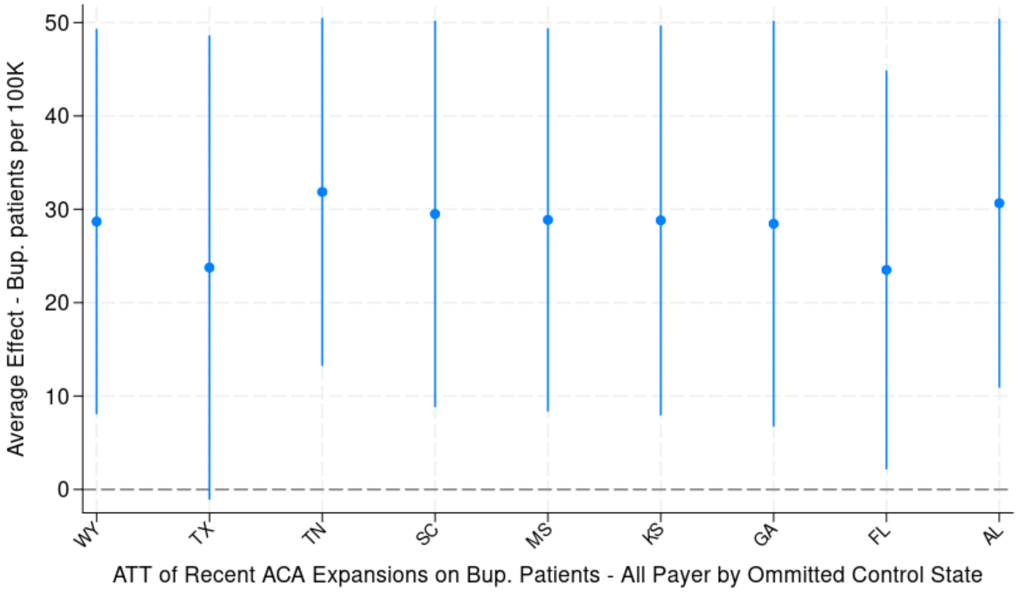

© 2026 Siegal N et al. *JAMA Network Open*.

eFigure 9. Medicaid Expansions and Buprenorphine Patients: Falsification Test Among Control States

Note: A plot of average treatment effects on the treated from staggered adoption difference-in-difference regressions, covering 24 months before and 24 months after the start of each Medicaid expansion, used to evaluate the rate of buprenorphine prescribed users per 100,000 of population in each state-month cell. Never-treated states were included in the control group, with one control state set as falsely treated for each treatment timing from the true treatment states for each specification, grouped by state falsely treated as noted below on the x axis. Data are from IQVIA LRx for 2013-2024, aggregating prescriptions for buprenorphine by state and month. Data for expansion dates from KFF and state populations from US Census Bureau. We limit the analysis in eFigure B9a to 2013-2018 and eFigure B9b to 2019-2024 to align with treatment timings from the main analysis (see Supplement eTable B1).

eFigure 9a: Early Medicaid Expansions

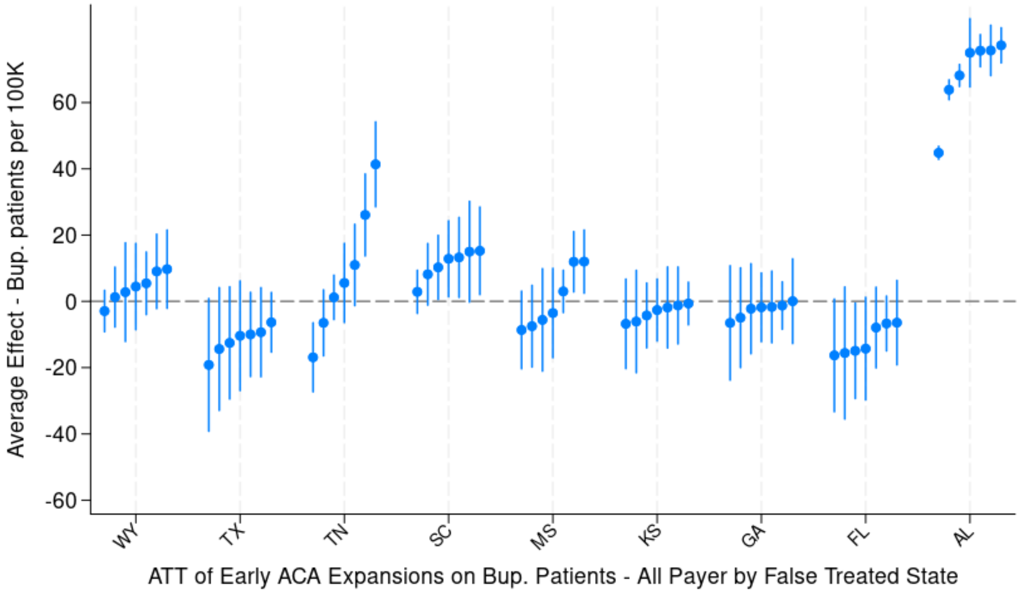

eFigure 8b: Recent Medicaid Expansions

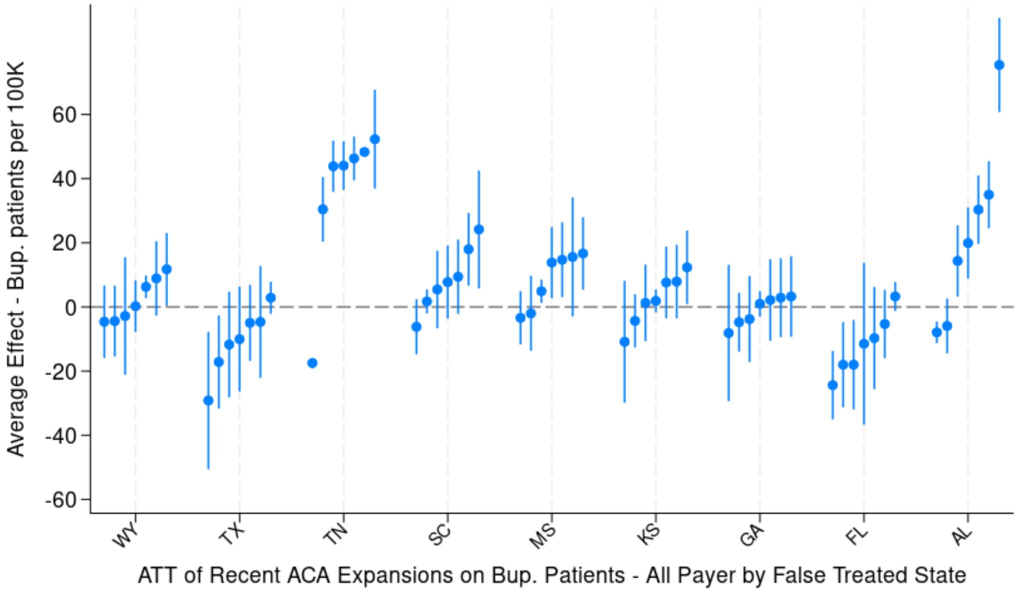

© 2026 Siegal N et al. *JAMA Network Open*.

Illustrative back-of-the-envelope estimate of potential overdose deaths averted

Our main analysis finds that in states that expanded Medicaid in 2019 or later, Medicaid expansion was associated with an average increase of 28.67 additional buprenorphine-treated patients per 100,000 residents (ATT). Applied to the approximate combined population of recent-expansion states (~40 million residents), this corresponds to roughly 11,500 additional individuals receiving buprenorphine treatment at any point in time:

$$28.67 \; per \; 100{,}000 \times 40{,}000{,}000 \approx 11{,}500 \; additional \; patients.$$

Numerous studies and reviews suggest that being maintained on buprenorphine is associated with a >50% reduction in overdose mortality among people with OUD.[17] For illustration, if we assume:

1. An annual overdose mortality risk of 2% among comparable individuals with untreated OUD, and
2. A 50–60% relative reduction in overdose mortality associated with buprenorphine treatment, then among 11,500 additional treated individuals, we would expect roughly:

$$11{,}500 \times 0.02 = 230 \; overdose \; deaths \; per \; year \; without \; treatment$$

and buprenorphine would avert approximately 50–60% of these, or about 115–140 overdose deaths per year in the recent-expansion states.

This calculation is intended solely as an illustrative, back-of-the-envelope estimate. It relies on strong assumptions about baseline overdose risk, duration and continuity of treatment, case mix among newly treated patients, and the external validity of mortality effect sizes drawn from prior studies to our population and time period. It should not be interpreted as a precise or causal estimate of lives saved, but rather as a way to contextualize the potential public health importance of the observed increases in buprenorphine treatment.

© 2026 Siegal N et al. *JAMA Network Open*.

eReferences.

1. Golan OK, Sheng F, Dick AW, et al. Differences in medicaid expansion effects on buprenorphine treatment utilization by county rurality and income: A pharmacy data claims analysis from 2009–2018. *Drug Alcohol Depend Rep*. 2023;9:100193. doi:10.1016/j.dadr.2023.100193

2. Knudsen HK, Hartman J, Walsh SL. The effect of Medicaid expansion on state-level utilization of buprenorphine for opioid use disorder in the United States. *Drug Alcohol Depend*. 2022;232:109336. doi:10.1016/j.drugalcdep.2022.109336

3. Olfson M, Zhang V (Shu), King M, Mojtabai R. Changes in Buprenorphine Treatment After Medicaid Expansion. *Psychiatr Serv*. 2021;72(6):633-640. doi:10.1176/appi.ps.202000491

4. Abraham AJ, Yarbrough CR, Harris SJ, Adams GB, Andrews CM. Medicaid Expansion and Availability of Opioid Medications in the Specialty Substance Use Disorder Treatment System. *Psychiatr Serv*. 2021;72(2):148-155. doi:10.1176/appi.ps.202000049

5. Choi S, Stein MD, Raifman J, Rosenbloom D, Clark JA. Estimating the impact on initiating medications for opioid use disorder of state policies expanding Medicaid and prohibiting substance use during pregnancy. *Drug Alcohol Depend*. 2021;229:109162. doi:10.1016/j.drugalcdep.2021.109162

6. Datta A, Oglesby W, George B. --Access to Health Insurance and Treatment Utilization for Opioid Use Disorder: Evidence From State Medicaid Expansions. *In Review*. Preprint posted online January 12, 2022. doi:10.21203/rs.3.rs-1220775/v1

7. Khatri UG, Howell BA, Winkelman TNA. Medicaid Expansion Increased Medications For Opioid Use Disorder Among Adults Referred By Criminal Justice Agencies: Study examines receipt of medications for opioid use disorder among individuals people referred by criminal justice agencies and other sources before and after Medicaid expansion. *Health Aff (Millwood)*. 2021;40(4):562-570. doi:10.1377/hlthaff.2020.01251

8. Meinhofer A, Witman AE. The role of health insurance on treatment for opioid use disorders: Evidence from the Affordable Care Act Medicaid expansion. *J Health Econ*. 2018;60:177-197. doi:10.1016/j.jhealeco.2018.06.004

9. Sharp A, Jones A, Sherwood J, Kutsa O, Honermann B, Millett G. Impact of Medicaid Expansion on Access to Opioid Analgesic Medications and Medication-Assisted Treatment. *Am J Public Health*. 2018;108(5):642-648. doi:10.2105/AJPH.2018.304338

10. Swartz N, Odayappan S, Chatterjee A, Cutler D. Impact of Medicaid expansion on inclusion of medications for opioid use disorder in homeless adults' treatment plans. *J Subst Use Addict Treat*. 2023;152:209059. doi:10.1016/j.josat.2023.209059

11. Wen H, Hockenberry JM, Borders TF, Druss BG. Impact of Medicaid Expansion on Medicaid-covered Utilization of Buprenorphine for Opioid Use Disorder Treatment. *Med Care*. 2017;55(4):336-341. doi:10.1097/MLR.0000000000000703

© 2026 Siegal N et al. *JAMA Network Open*.

12. Saloner B, Levin J, Chang HY, Jones C, Alexander GC. Changes in Buprenorphine-Naloxone and Opioid Pain Reliever Prescriptions After the Affordable Care Act Medicaid Expansion. *JAMA Netw Open*. 2018;1(4):e181588. doi:10.1001/jamanetworkopen.2018.1588

13. Shakya S, Harris SJ. Medicaid expansion and opioid supply policies to address the opioid overdose crisis. *Drug Alcohol Depend Rep*. 2022;3:100042. doi:10.1016/j.dadr.2022.100042

14. Callaway B, Sant'Anna PHC. Difference-in-Differences with multiple time periods. *J Econom*. 2021;225(2):200-230. doi:10.1016/j.jeconom.2020.12.001

15. Sun L, Abraham S. Estimating dynamic treatment effects in event studies with heterogeneous treatment effects. *J Econom*. 2021;225(2):175-199. doi:10.1016/j.jeconom.2020.09.006

16. Gardner J. Two-stage differences in differences. *arXiv*. Preprint posted online 2022. doi:10.48550/ARXIV.2207.05943

17. F J, Mueller PP, Chen Q, et al. Estimated Reductions in Opioid Overdose Deaths With Sustainment of Public Health Interventions in 4 US States. *JAMA Netw Open*. 2023;6(6):e2314925. doi:10.1001/jamanetworkopen.2023.14925

© 2026 Siegal N et al. *JAMA Network Open*.
