# Supplementary material for: Medicaid Expansion and Buprenorphine Dispensing in Early vs Recent Expansion States
Source: JAMA Netw Open. 2026 Feb 18;9(2):e2559803. doi: 10.1001/jamanetworkopen.2025.59803 (PMC12917678; doi:10.1001/jamanetworkopen.2025.59803)
Supplement: Supplement 2. — Data Sharing Statement [file jamanetwopen-e2559803-s002.pdf]

Data Sharing Statement

Siegal. Medicaid Expansion And Buprenorphine Dispensing In Early vs Recent Expansion States. *JAMA Netw Open*. Published February 18, 2026.
doi:10.1001/jamanetworkopen.2025.59803

Data
Data available: No
